# *Nicotiana tabacum* Kunitz Peptidase Inhibitor-like Protein Regulates Intercellular Transport

**DOI:** 10.3390/plants14192955

**Published:** 2025-09-23

**Authors:** Natalia M. Ershova, Ekaterina V. Sheshukova, Alfiya R. Alimova, Kamila A. Kamarova, Eugene A. Arifulin, Tatiana V. Komarova

**Affiliations:** 1 Vavilov Institute of General Genetics, Russian Academy of Sciences, 119991 Moscow, Russia; ershova@vigg.ru (N.M.E.); sheshukova@vigg.ru (E.V.S.); alimovaar@my.msu.ru (A.R.A.); kamila.kamarova@yandex.ru (K.A.K.); 2 Faculty of Bioengineering and Bioinformatics, Lomonosov Moscow State University, 119991 Moscow, Russia; 3 Belozersky Institute of Physico-Chemical Biology, Lomonosov Moscow State University, 119991 Moscow, Russia; lewanit@gmail.com

**Keywords:** Kunitz peptidase inhibitor-like protein (KPILP), tobacco mosaic virus, potato virus X, intercellular movement, chloroplast retrograde signaling, antiviral defense, plant-virus interactions

## Abstract

A coordinated and generalized plant response to adverse environmental factors largely depends on the proper and finely-tuned regulation of intercellular transport via plasmodesmata (PD). However, the knowledge of the whole network of PD-controlling mechanisms is far from complete. Earlier, a cellular factor, Kunitz peptidase inhibitor-like protein (KPILP), that affects PD gating and plays a proviral role, was identified in *Nicotiana benthamiana* plants. Here we characterized its homolog from *N. tabacum*, *NtKPILP*, which is hardly detectable in leaves of intact plants, in contrast to roots, flowers and seeds where *NtKPILP* is highly expressed. However, its mRNA accumulation in leaves increases in response to various stresses, including viral infection. *NtKPILP* was demonstrated to affect chloroplast functioning. Using the virus-induced gene silencing approach, we have shown that *NtKPILP* downregulation negatively affects intercellular transport of macromolecules, inducing callose deposition at PD and suppressing beta-1,3-glucanase mRNA accumulation. Together, the obtained results indicate that NtKPILP is a viral infection-responsive cellular factor that is involved in PD permeability regulation, sharing thus the features of KPILPs from other *Nicotiana* species.

## 1. Introduction

External stimuli of biotic and abiotic nature lead to the activation of various genes involved in plant adaptation to stresses. The rise of signals in the cell, the launch of defense mechanisms and signal spread throughout the organism occur, among other things, through plasmodesmata (PD)—unique for plants, cellular channels connecting protoplasts of the adjacent cells. These structures mediate the flux of ions and water and transport of such compounds as sugars and phytohormones, as well as macromolecules (transcription factors, non-cell autonomous proteins, regulatory and even messenger RNAs) [1,2,3]. Plant viruses exploit PD for spread throughout the plant [4]. PD permeability is strictly regulated, and several major mechanisms are known to be involved in PD control. One of the most studied mechanisms is based on callose deposition around PD. Callose is a cell wall polysaccharide that accumulates around the plasmodesmata channel, which leads to a decrease in PD permeability. Callose-mediated plasmodesmata aperture reduction is one of the mechanisms regulating intercellular transport. This mechanism is based on the coordinated work of enzymes that synthesize (callose synthases) and degrade (beta-glucanases) callose [5]. In addition to these two groups of enzymes, many other cellular factors localized both in PD and in other cellular compartments participate in the regulation of callose levels [3,5,6,7], and many more components of callose metabolism regulation during growth, development, and exposure to stress factors are still being discovered [8,9,10,11]. The achievements of recent years demonstrated the importance of PD as hubs and key players in hormonal signaling [3]. In addition to factors involved in structural and functional changes in response to various stimuli and localized at PD, there is growing evidence that the PD permeability is controlled by signals from other organelles, primarily from chloroplasts and mitochondria [12,13,14,15]. Chloroplasts are very dynamic photosynthetic organelles that play an active role in antiviral defense, being a source of various metabolites and phytohormones, including salicylic and jasmonic acids, as well as reactive oxygen species [16,17,18]. Metabolites synthesized in chloroplasts serve as signals transferred to the nucleus, thereby regulating the expression of various genes and determining the physiological state of the cell. Such a regulatory network is designated as chloroplast retrograde signaling (CRS) system [19]. Intercellular transport could also be regulated via CRS. The organelles–nucleus–PD signaling model (ONPS) describes and summarizes such regulation pathways based on the signal transfer from chloroplasts and mitochondria to the nucleus, affecting there expression of genes involved in PD permeability control [13,14].

Genes associated with photosynthesis are targets for many phytoviruses. Viral factors are able to interfere with the biosynthesis of proteins involved in chloroplasts’ functioning at the level of their genes’ transcription, post-transcriptional regulation, translation, and transport [20,21]. Moreover, the 126 kDa protein of the tobacco mosaic virus (TMV) replication complex binds to the following chloroplast proteins involved in antiviral defense: PsbO (a subunit of the oxygen-evolving complex of PSII) [22], RuBisCo activase (RCA), and γ-subunit of ATP synthase (AtpC) [23]. PsbO-encoding gene silencing, similar to inhibition of PSII, was shown to stimulate TMV reproduction, while TMV infection per se led to a decrease in *PsbO* mRNA accumulation. It was hypothesized that PsbO binding to the TMV 126kDa protein hampers its interaction with the 183 kDa replicase protein [22]. Silencing of the RCA- and AtpC-encoding genes resulted in stimulation of TMV infection, indicating that they play an antiviral role; however, the particular mechanism is unclear [23]. The small subunit of RuBisCo (RbCS) binds the TMV movement protein (MP) and negatively affects viral intercellular transport. In addition, *rbcS* silencing increases plant susceptibility to TMV, which is accompanied by low expression of the pathogenesis-related (PR)-1a gene from the group of PR genes responsible for defense against pathogens [24].

Numerous genes are activated in response to various stress factors: penetration of bacteria, fungi, viruses, and insects. Among them are genes encoding one of the groups of PR proteins—protease inhibitors (PIs), which include several protein families [25,26]. The members of the Kunitz protease inhibitors (KPIs) family possess the specificity towards serine proteases [25]. In addition to the “true” KPIs, there are numerous KPI-like proteins (KPILP) that have most of the KPIs’ structural characteristic features but lack the conserved amino acid residues in the active center necessary for functioning as inhibitors [27]. Previously, a transcriptome analysis of *N. benthamiana* plants infected with TMV revealed a high level of mRNA encoding one of such KPILPs, namely NbKPILP (GeneBank Ac. D4IHB9). NbKPILP contains elements that are characteristic of KPI family members: the Kunitz motif, KPI reactive loop and two pairs of cysteine residues forming disulfide bonds. However, its predicted active center lacks amino acid residues responsible for interaction with trypsin, unlike the soybean Kunitz-type trypsin inhibitor and the KPI of *A. thaliana*; moreover, NbKPILP was experimentally demonstrated not to exhibit inhibitory activity towards trypsin [27]. The recent studies of NbKPILP functioning revealed its important role in responses to abiotic (prolonged light deprivation) [27] and biotic (viral infection) [28,29] stresses. It was shown that upon viral infection, NbKPILP participates in the regulation of CRS by suppressing the expression of nuclear genes encoding chloroplast proteins and determining the physiological state of these organelles. In addition, increased *NbKPILP* expression leads to stimulation of intercellular transport in a callose-dependent mode [28,29].

Putative and confirmed KPILPs have been found in several species of the *Solanaceae* family based on sequence similarity [27]. However, an additional verification should be performed before extrapolation of the features and functions of the studied gene/protein from the model plant to its homologs from other, even closely related species and different cultivars of the same plant species. In the current work, we characterize NtKPILP, the *N. tabacum* homolog of NbKPILP. We demonstrated that *NtKPILP* expression increases in response to long-term plant incubation in the darkness and infection with two taxonomically different viruses. *NtKPILP* upregulation leads to the suppression of antiviral response genes, as well as CRS marker genes. *NtKPILP* downregulation results in the reduction of the intercellular transport of macromolecules, affecting PD callose deposition and β-1,3-glucanase-endcoding mRNA accumulation. Thus, based on the obtained results, it could be concluded that KPILP exhibits similar features and is likely to perform the same functions in different tobacco species—*N. benthamiana* and *N. tabacum*.

## 2. Results

### 2.1. Nicotiana tabacum KPILP Identification and Expression Level Analysis

**Figure 1:**
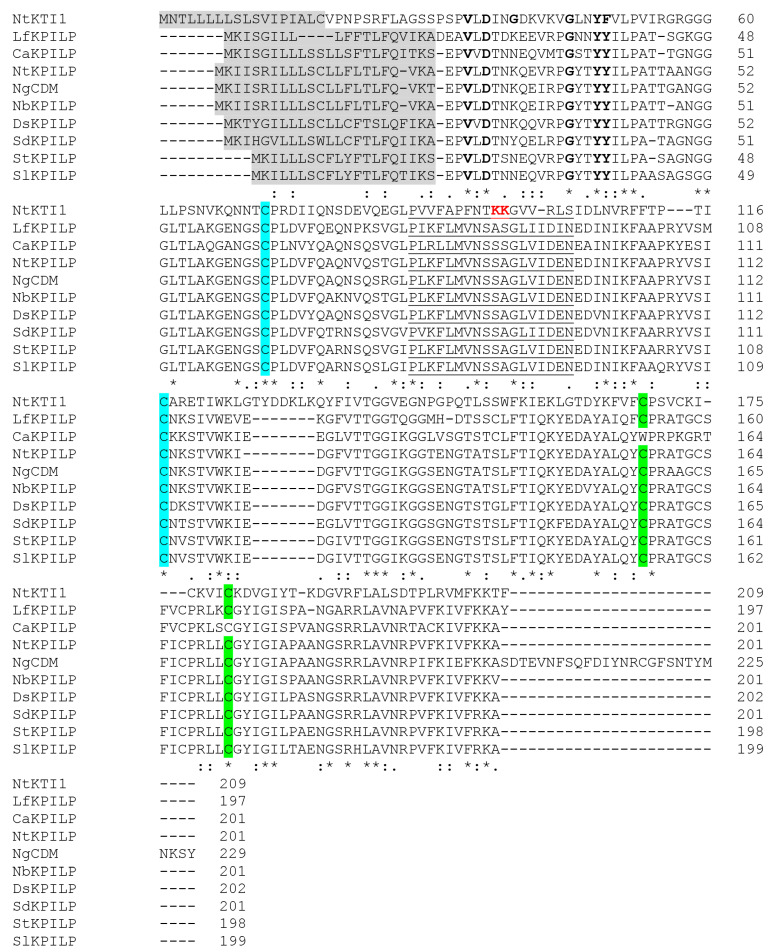
**Multiple alignment of various KPILPs from the members of *Solanaceae* family**. The signal peptide is highlighted in grey, black bolded letters stand for the main residues of the Kunitz motif, the amino acids comprising the reactive loop of KPIs are underlined, P and P′ sites responsible for enzymatic activity are bolded and red. The pairs of cysteine residues forming the disulfide bonds are highlighted in blue and green, respectively. (*) indicate fully conserved residue, (:) indicate similar residues, (.) indicate that one of the ‘weaker’ groups is fully conserved. NtKTI1 (GenBank Ac. FJ494920), which possesses confirmed inhibitor activity [30], is shown for comparison. Sequences of KPILP from *S. lycopersicum* (SlKPILP) (Solyc03g098740.1.1) and *Capsicum annuum* KPILP (CaKPILP) (CA03g23560) are obtained from the SolGenomics database. Sequences of *N. benthamiana* KPILP (NbKPILP) (D4IHB9), *N. glutinosa* biotic cell death-associated protein (NgCDM) (Q850R9), *S. dulcamara* (SdKPILP) (XP_055826690.1), *Datura stramonium* hypothetical protein HAX54_027716 (DtKPILP) (MCD9641504.1), *Lycium ferocissimum* KPILP (LfKPILP) (XP 059304499.1), and *S. tuberosum* (StKPILP) (XP 006353918.1) are obtained from GenBank. The alignment was performed using ClustalW.

Based on the amino acid sequence similarity with previously identified proviral factor from *Nicotiana benthamiana*, NbKPILP, an unannotated sequence, further designated as NtKPILP, was retrieved from *Nicotiana tabacum* genome (SolGenomics database https://solgenomics.net/organism/Nicotiana_tabacum/genome, accessed 15 July 2022). The degree of identity between *NbKPILP* and predicted *NtKPILP* nucleotide sequences was 95%, while the amino acid similarity of the encoded proteins was 96%. The multiple alignment of *N. tabacum* KPILP and KPILPs from other members of the Solanaceae family, including tobacco species (*N. benthamiana* and *N. glutinosa*), *Solanum lycopersicum*, *S. tuberosum*, *S. dulcamara*, *Capsicum annuum*, *Datura stramonium*, and *Lycium ferocissimum*—showed that all of them contain signal peptide and the main signatures of KPIs (Figure 1). However, the amino acid residues forming the active site in most of the KPIs—presumably Lys or Arg—are absent in the analyzed KPILPs.

**Figure 2:**
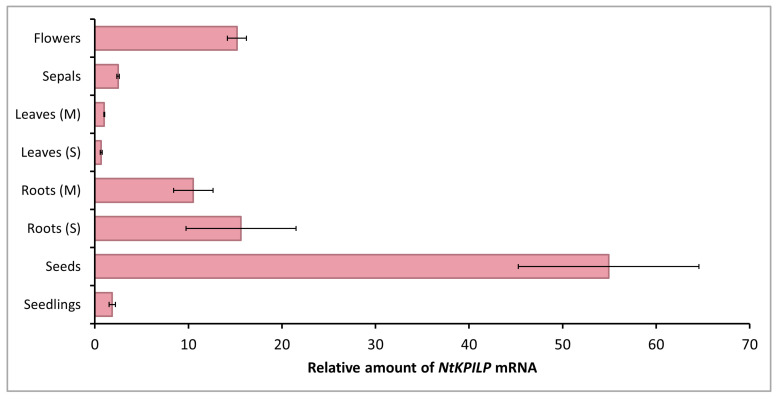
**Quantitative RT-PCR analysis of *NtKPILP* mRNA levels in different parts of *N. tabacum* (cv. Petit Havana) plants**. Leaves and roots were harvested from 5–6-week-old plants (M, mature plants) or seedlings (S). The levels of expression are normalized to the *PP2A* gene. The *NtKPILP* expression level in leaves (M) was taken as 1. The plot represents the mean values and standard error (SE) of five biological replicates.

It is known that in *N. benthamiana* plants, the level of *NbKPILP* mRNA accumulation differs significantly in roots and leaves: a 1000-fold excess of the corresponding mRNA accumulation level was detected in roots compared with leaves [27]. In the current work, an analysis of the *NtKPILP* expression level in different organs of intact tobacco plants (Figure S1) was performed. *NtKPILP* mRNA was clearly detected in the roots and leaves of both mature plants (5–6 weeks old) and seedlings with two leaves, as well as in stems, flowers, sepals, and seeds (Figure 2).

However, *NtKPILP* mRNA content in leaves was the lowest. The relative amount of *NtKPILP* mRNA in roots appeared to be 10–15 times higher than in the leaves. Surprisingly, the flowers also showed a 15-fold increase in *NtKPILP* expression compared with leaves. In addition, high levels of *NtKPILP* mRNA accumulation were detected in seeds.

Thus, similar to *N. benthamiana KPILP*, *NtKPILP* is highly expressed in roots and poorly expressed in mature leaves.

### 2.2. Incubation in Darkness and Systemic Viral Infection Stimulate NtKPILP mRNA Accumulation in Leaves

**Figure 3:**
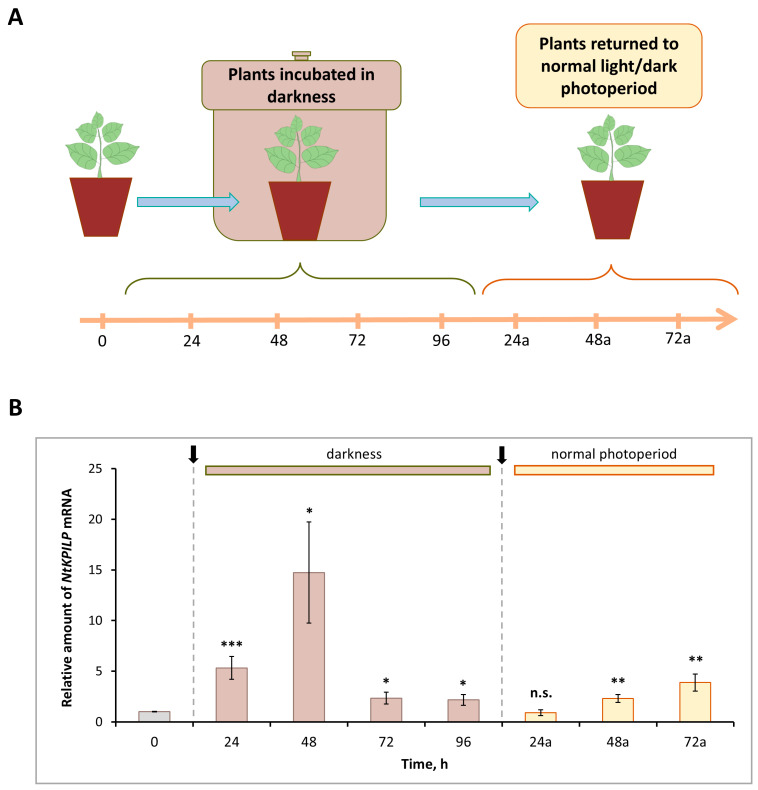
***NtKPILP* mRNA accumulation is induced in response to plant incubation without light**. (**A**) Schematic representation of experimental workflow, with sample collecting time points indicated. (**B**) Relative amount of *NtKPILP* mRNA in leaves of *N. tabacum* (cv. Petit Havana) plants incubated in darkness and returned to normal light/dark photoperiod as determined with qRT-PCR. The amount of *NtKPILP* mRNA at time point “0” is taken as 1. Three independent experiments with three biological repeats each were performed. Paired two-tailed Student’s *t*-test was applied to assess statistical significance of difference between the samples harvested at “0” time point and other time points, *, *p* < 0.05; **, *p* < 0.01; ***, *p* < 0.001; n.s., not significant.

Previously, it was demonstrated that *N. benthamiana KPILP* mRNA accumulation significantly increased in leaves of the plants incubated in the darkness for the period from 48 to 96 h (h) [27]. Here, we analyzed the effect of such an incubation on *NtKPILP* expression. Plants were kept without light for 96 h, and every 24 h, the samples were collected (Figure 3A). In addition, samples were harvested 24, 48, and 72 h after plants were returned to the normal 16h/8h light/dark photoperiod.

Similar to *NbKPILP*, *NtKPILP* appeared to be induced by prolonged darkness (Figure 3B). However, *NtKPILP* mRNA accumulation started to increase earlier, by 24 h of incubation without light. The mRNA level was 6.7-fold higher compared with the “zero” control time-point, and by 48 h, it peaked, showing an 18-fold increase, followed by a decrease at the 96 h time point (Figure 3B). Notably, 24 h after (24a-timepoint) restoration of the normal 16/8 light/dark regime, *NtKPILP* mRNA amount returned to the initial level.

Obviously, such light deprivation affects photosynthesis. Thus, the expression of genes related to photosynthesis was assessed in that experiment. Among them were genes encoding RBCS1A (small subunit isoform of RuBisCO), HEMA1 (glutamyl-tRNA reductase involved in tetrapyrrole biosynthesis), LHCB21 (chloroplast light-harvesting complex isoform 21), RCA (RuBisCO activase), and AtpC (ATP synthase γ-subunit). Indeed, mRNA levels for these genes were significantly decreased during plant incubation in the darkness (Figure S2). Moreover, a correlation between darkness-mediated *NtKPILP* upregulation and photosynthesis-associated genes downregulation could be observed.

**Figure 4:**
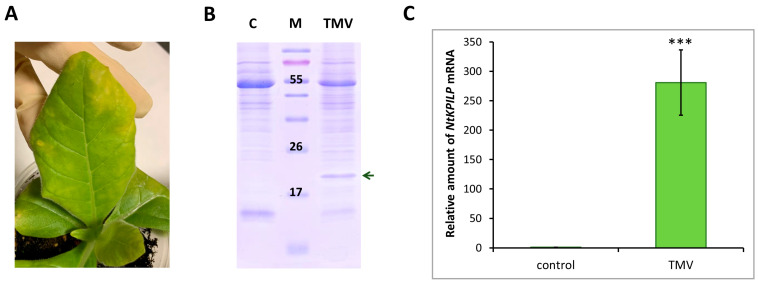
**TMV induces *NtKPILP* mRNA accumulation**. (**A**) Symptoms on *N. tabacum* (cv. Petit Havana) leaves infected with TMV on the 7–10 dpi. (**B**) Analysis of total soluble protein from leaves with systemic infection induced by TMV and control plants in a 15% polyacrylamide gel, followed by Coomassie staining. The band corresponding to TMV CP is indicated with a dark green arrow. M: protein molecular weight markers, C: mock-inoculated control. (**C**) Relative amount of *NtKPILP* mRNA in the mock-inoculated (control) and TMV-infected plants as determined with qRT-PCR. The level of mRNA accumulation for the control was taken as 1. Mean values and SE were calculated from five biological replicates. The difference between control and samples from TMV-infected plants is significant: ***, *p* < 0.001 (paired two-tailed Student’s *t*-test).

It was previously shown that in intact *N. benthamiana* leaves, *NbKPILP* mRNA accumulation is suppressed as a result of expression of the alternative nested reading frame, encoding a 53-amino acid peptide [27]. However, infection with the crTMV:GFP viral vector based on the crTMV genome, as well as TMV, crTMV, and potato virus X (PVX) systemic infection [28,29], led to a significant increase in the *NbKPILP* mRNA level in *N. benthamiana* plants. Systemic TMV infection of *N. tabacum* (cv. Samsun nn) plants, as well as TMV-induced hypersensitive response (HR) in *N. glutinosa*, was also accompanied by activated *KPILP* expression [27,31]. In order to study *NtKPILP* expression pattern upon viral infection, *N. tabacum* (cv. Petit Havana) plants were infected with viruses from two different families: TMV (family *Virgaviridae*) and PVX (family *Alphaflexiviridae*). In the first series of experiments, the plants were divided into two groups: mock-inoculated and TMV-infected. Each group contained five plants. Systemic TMV infection was confirmed by the presence of symptoms on the leaves (Figure 4A), as well as by the presence of the band corresponding to TMV coat protein (CP) after separation of proteins from the infected leaf extract in polyacrylamide gel (PAAG) (Figure 4B). Leaves of the mock-inoculated plants were used as a negative control. Symptoms of TMV infection on *N. tabacum* (cv. Petit Havana) plants differ from the symptoms characteristic of *N. tabacum* (cv. Samsun nn): instead of “classic” mosaic, infected leaves demonstrate altered pigmentation (Figure 4A). Seven to ten days post-infection, samples from the leaves of these two groups were collected and total RNA extracted. Quantitative RT-PCR revealed a 270-fold increase in *NtKPILP* mRNA accumulation in plants with TMV systemic infection compared with the mock-inoculated plants (Figure 4C).

Thus, *NtKPILP* expression is upregulated in response to TMV infection in *N. tabacum* (cv. Petit Havana), i.e., *KPILP* is a TMV-inducible cellular factor in both *N. benthamiana* and *N. tabacum*.

**Figure 5:**
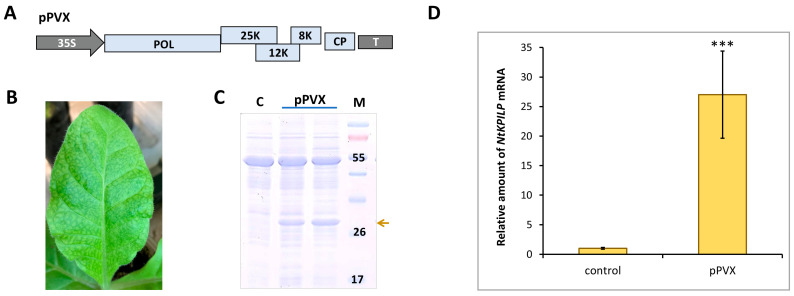
**PVX infection induces *NtKPILP* mRNA accumulation**. (**A**) Schematic representation of PVX-based vector pPVX. 35S: *Cauliflower mosaic virus* 35S promoter; T: 35S terminator of transcription, POL: gene encoding PVX RNA-dependent RNA polymerase; 25K, 12K, 8K: triple gene block encoding PVX movement proteins; CP: PVX coat protein encoding gene; (**B**) Symptoms on *N. tabacum* (cv. Petit Havana) leaves infected with PVX on 10–14 dpi. (**C**) Analysis of total soluble proteins from leaves with PVX systemic infection and control mock-inoculated plants in PAAG, followed by Coomassie staining. Bands corresponding to PVX CP are indicated with a yellow arrow, M: protein molecular weight markers, and C: control (mock-inoculated). (**D**) Relative amount of *NtKPILP* mRNA in the mock-inoculated (control) and PVX-infected plants as determined using qRT-PCR. The level of mRNA accumulation for the control was taken as 1. Mean values and SE were obtained from four independent experiments with five biological replicates in each. The difference between control and samples from PVX-infected plants is significant: ***, *p* < 0.001 (paired two-tailed Student’s *t*-test).

To check if *NtKPILP* mRNA accumulation increases in response to other viruses, the analysis of *N. tabacum* (cv. Petit Havana) plants infected with potato virus X (PVX) was performed. Plants were agroinfiltrated with PVX-encoding viral vector (Figure 5A). Successful systemic infection was confirmed by the development of characteristic symptoms 10–14 days later (Figure 5B), and the presence of PVX CP in the leaf extract (Figure 5C). Assessment of *NtKPILP* mRNA levels in leaves with systemic PVX infection showed a 27-fold increase compared with the mock-inoculated plants (Figure 5D).

Thus, *NtKPILP* mRNA accumulation is significantly increased in response to both TMV and PVX, i.e., taxonomically distinct viruses.

### 2.3. Downregulation of NtKPILP Expression in Nicotiana tabacum Plants

**Figure 6:**
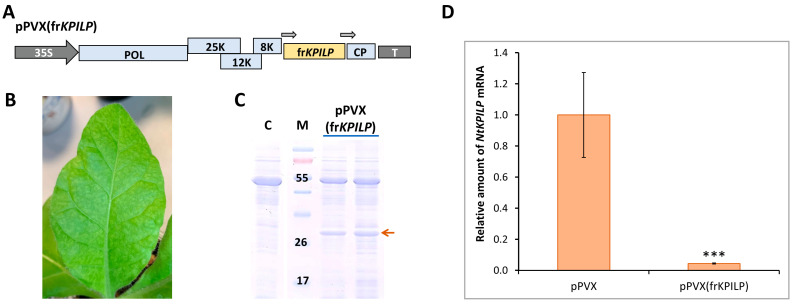
***NtKPILP* virus-induced gene silencing**. (**A**) Schematic representation of PVX-based vector pPVX(frKPILP). Arrows indicate the PVX subgenomic CP gene promoter. 35S: *Cauliflower mosaic virus* 35S promoter; T: 35S terminator of transcription, POL: gene encoding PVX RNA-dependent RNA polymerase; 25K, 12K, 8K: triple gene block encoding PVX movement proteins; CP: PVX coat protein encoding gene (**B**) Symptoms on *N. tabacum* (cv. Petit Havana) leaves infected with pPVX(frKPILP) on 10–14 dpi (**C**) Analysis of total soluble protein from leaves with systemic infection induced with pPVX(frKPILP) and control mock-inoculated plants in PAAG followed by Coomassie staining. Bands corresponding to PVX CP are indicated with the red arrow. M: protein molecular weight markers, C: mock-inoculated control. (**D**) Relative amount of *NtKPILP* mRNA in the pPVX- or pPVX(frKPILP)-infected plants as determined by qRT-PCR. The level of mRNA accumulation for pPVX-infected plants was taken as 1. Mean values and SE were obtained from four independent experiments with five biological replicates in each. The difference between samples from PVX- or pPVX(frKPILP)-infected plants is significant: ***, *p* < 0.001 (paired two-tailed Student’s *t*-test).

An approach based on the virus-induced gene silencing (VIGS) was used to study the effects of *NtKPILP* downregulation. Since the sequences of *NbKPILP* and *NtKPILP* have a similarity of 95% (Figure S3), the previously obtained pPVX(frKPILP) vector (Figure 6A) based on the PVX genome with an *NbKPILP* fragment (frKPILP) insert [28] was used to induce *NtKPILP* silencing. To avoid off-target silencing, the frKPILP was analyzed using the VIGS tool (https://vigs.solgenomics.net/, accessed 20 August 2022); no additional target sequences were revealed. Notably, the imperfect match of frKPILP used for silencing to *NtKPILP* sequence should not hinder, but, on the contrary, likely promotes more effective silencing as was demonstrated for other target genes [32].

To induce *NtKPILP* silencing, a group of five plants was used. pPVX(frKPILP) vector was delivered to the cells of lower leaves via agroinfiltration. Characteristic symptoms of PVX infection were detected on the upper leaves of *N. tabacum* by the 10–14th dpi (Figure 6B). Total soluble protein in the leaf extracts of the studied plants was analyzed. PVX CP was detected in the extracts of upper leaves (Figure 6C).

On the 14th dpi, samples were collected from leaves with confirmed systemic PVX infection to extract total RNA and analyze the relative level of *NtKPILP* mRNA accumulation. In this series of experiments, a group with systemic PVX infection (agroinfiltrated with pPVX vector, Figure 5A) was used as a control. In addition, a group of mock-inoculated plants of the same age was analyzed. The results of qRT-PCR revealed that *NtKPILP* expression was ca. 27-fold lower in the leaves of plants infected with pPVX(frKPILP) compared with the pPVX-inoculated plants (Figure 6D). Notably, the assessment of PVX RNA accumulation showed no significant difference between the reproduction of both viral vectors, pPVX and pPVX(frKPILP) (Figure S4), indicating that regardless of the presence of the frKPILP insert, the viral vector functions correctly, and the induction of *NtKPILP* silencing does not affect the efficiency of PVX infection in *N. tabacum*.

Thus, a model system of *N. tabacum* (cv. Petit Havana) plants with *NtKPILP* knockdown was created.

### 2.4. NtKPILP Is Involved in the Regulation of Chloroplast Functioning and Antiviral Defense Response in N. tabacum Leaves

**Figure 7:**
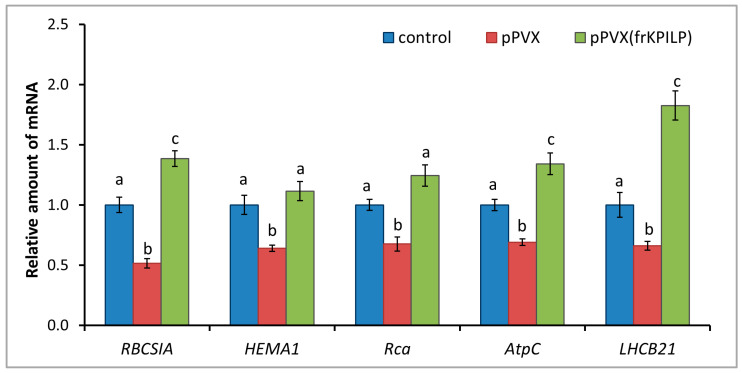
**NtKPILP affects the expression of photosynthesis-associated nuclear-encoded genes.** Relative mRNA level of CRS marker genes and genes involved in antiviral defense in plants with systemic PVX or PVX(frKPILP) infection as determined using qRT-PCR. The level of mRNA accumulation for each gene in mock-inoculated (control) plants was taken as 1. Mean values and SE were obtained from four independent experiments with five biological replicates in each. The data was analyzed using ANOVA. Bars without similar letters indicate significant differences according to Tukey HSD at *p* < 0.05, while bars with shared letters are not significantly different.

KPILP was previously shown to affect chloroplast retrograde signaling (CRS) and carbon metabolism in *N. benthamiana* infected with PVX [28,29]. To test whether *N. tabacum* KPILP has similar properties, the following experimental system was used: the expression levels of marker genes encoding proteins involved in CRS regulation and antiviral defense responses were assessed. Among them are genes for RBCS1A, HEMA1 (glutamyl-tRNA reductase involved in tetrapyrrole biosynthesis (CRS mediator)), LHCB21, RCA, and AtpC. RCA and AtpC are involved in plant defense reactions, especially in the antiviral response [23]. Three groups of plants were compared: mock-inoculated (“control”), “pPVX” group with increased *NtKPILP* expression (i.e., with systemic PVX infection that induces ca. 27-fold *NtKPILP* upregulation), and “pPVX(frKPILP)” group with *NtKPILP* knockdown. Activated *NtKPILP* expression led to suppression of the analyzed genes compared with the “control” group. In contrast, *NtKPILP* knockdown resulted in an increase in their mRNA accumulation up to the level of “control” or even exceeding it (Figure 7).

Therefore, the obtained results could indicate NtKPILP involvement in the regulation of CRS and antiviral defense responses.

### 2.5. NtKPILP Regulates Intercellular Transport of Macromolecules via a Callose-Dependent Mechanism

**Figure 8:**
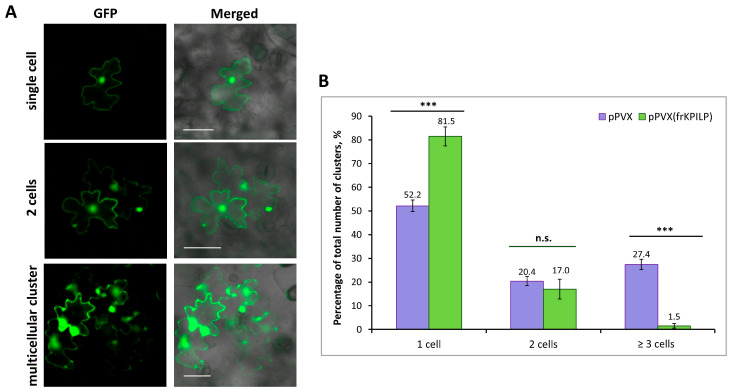
***NtKPILP* downregulation suppresses intercellular transport of macromolecules.** (**A**) Visualization of a single cell, group of two cells or multicellular cluster containing 2×GFP in epidermal cells of *N. tabacum* (cv. Petit Havana) leaves. Bar = 50 µm. (**B**) Quantification of 2×GFP intercellular movement in plants with *NtKPILP* silencing (pPVX(frKPILP)-infected) compared with control pPVX-infected plants. Mean values and SE are presented. Three experiments with at least three biological replicates each were performed, and at least 200 cell clusters per one infiltration area were analyzed. Paired two-tailed Student’s *t*-test was applied to assess the statistical significance of the difference between plants of the two groups. ***: *p* < 0.001, n.s.: difference is not statistically significant.

It was previously shown that increased *NbKPILP* expression affects intercellular transport of macromolecules, including TMV and crTMV local spread in *N. benthamiana* [28,29]. NbKPILP was hypothesized to be one of the units of the organelle–nucleus–PD signaling (ONPS) pathway regulating PD size exclusion limit upon viral infection. To assess the effect of *NtKPILP* expression on the efficiency of intercellular transport, the 2×GFP reporter molecule, which represents two fused copies of GFP, was used. 2×GFP can spread from the primary transformed cell to neighboring cells only when PD are dilated. Thus, the formation of GFP-containing clusters of several cells indicates an increase in PD permeability and activation of intercellular transport (Figure 8A). *N. tabacum* (cv. Petit Havana) plants with systemic pPVX or pPVX(frKPILP) infection and confirmed up- or downregulated *NtKPILP* expression, as well as plants of the mock-inoculated “control” group, were infiltrated with a suspension of agrobacteria to deliver a plasmid encoding 35S-2×GFP into leaf cells. In the “control” group of *N. tabacum* plants 48 h after agroinfiltration, 100% of the cells with a fluorescent GFP signal were represented with single cells; 2×GFP did not move from cell to cell, since the PD in intact leaves do not allow proteins larger than 47 kDa to pass through. In plants inoculated with PVX-based vectors, the formation of multicellular (consisting of three or more cells) clusters containing GFP signal was observed (Figure 8B). In these two groups, 2×GFP cell-to-cell movement was possible due to active virus (PVX) infection. However, the proportion of clusters of different sizes varied between the groups. In plants of the “pPVX” group, the number of multicellular clusters increased up to 27.4% (±2.2), and single-cell clusters accounted for 52.2% (±2.5) of the total number of clusters. On the contrary, in leaves with suppressed *NtKPILP* expression (“pPVX(frKPILP)” group), the ratio of clusters consisting of 3 or more cells was only 1.5% (±1), and single-cell clusters constituted 81.5% (±4). Therefore, suppression of *NtKPILP* expression leads to a decrease in 2×GFP intercellular transport activity.

One of the mechanisms of PD permeability regulation involves the synthesis and degradation of callose in the PD region. When PD callose is degraded, the PD aperture increases, while callose deposition has the opposite effect [7].

**Figure 9:**
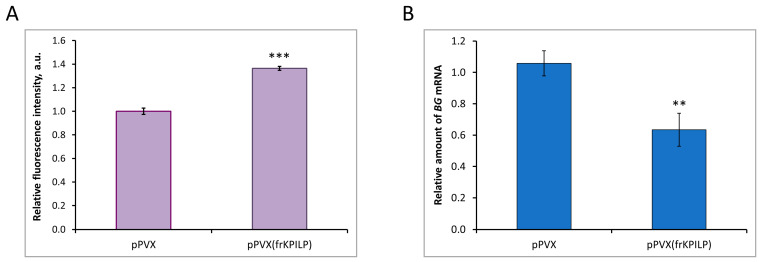
***NtKPILP* affects PD callose deposition**. (**A**) Relative PD callose levels in *N. tabacum* (cv. Petit Havana) with *NtKPILP* silencing (pPVX(frKPILP)-infected) compared with control pPVX-infected plants as estimated with measurement of aniline blue-stained callose fluorescence intensity. The level of callose in pPVX-infected plants was taken as 1. Mean values in arbitrary units (a.u.) and SE are presented. Four biological replicates were performed for each group; no less than eight areas from each leaf were analyzed, resulting in at least 1000 fluorescent dots in total quantified. (**B**) Relative amount of *BG* mRNA in the same leaves as in the panel (**A**), as assessed using qRT-PCR. Mean values and SE were obtained from four biological replicates for each group. In both panels, the difference between samples from PVX- or pPVX(frKPILP)-infected plants is significant: **, *p* < 0.01, ***, *p* < 0.001 (paired two-tailed Student’s *t*-test).

To reveal the mechanism underlying NtKPILP effects on the intercellular transport in *N. tabacum* (cv. Petit Havana) leaves, the assessment of PD callose levels upon *NtKPILP* knockdown was performed. Leaves in two groups of plants (infected with pPVX or pPVX(frKPILP)) were infiltrated with an aniline blue solution, after which the fluorescence of the stained callose was visualized using a confocal fluorescence microscope (Figure S5). The average intensity of callose fluorescence was calculated according to the protocol described by Zavaliev and Epel [33]. In pPVX(frKPILP) plants with suppressed *NtKPILP* expression, the callose level was 40% higher than in pPVX plants (Figure 9A). Thus, when *NtKPILP* is downregulated, the PD callose level increases, leading to a reduction in PD permeability. To further clarify the molecular mechanism underlying *NtKPILP* effects on intercellular transport and callose deposition, we assessed the mRNA level of beta-1,3-glucanase (BG). BG is one of the key enzymes of callose metabolism; it catalyzes callose hydrolysis in the cell wall, leading to a reduction in PD callose depositions [34,35]. In plants with *NtKPILP* knockdown, *BG* expression level was halved compared with plants from the control group (Figure 9B). Therefore, *NtKPILP* downregulation led to a decrease in *BG* mRNA accumulation and an increase in PD callose deposition. We concluded that NtKPILP regulates PD permeability, affecting callose deposits via modulation of *BG* expression.

Taken together, results on *NtKPILP* effects on intercellular transport and callose depositions indicate that *NtKPILP* regulates PD aperture via a callose-dependent mechanism modulating *BG* expression, thereby affecting cell-to-cell transport of macromolecules (including 2×GFP).

## 3. Discussion

Plants are constantly exposed to adverse environmental effects during their growth and development, facing many stress factors of abiotic (temperature fluctuations, water deficiency, high salt concentrations, etc.) and biotic (bacteria, viruses, fungi, etc.) origin. Stress, in particular biotic (pathogen attack), triggers defense reactions accompanied by the activation of cascades of various genes. Previously, during the transcriptome analysis of TMV-infected *N. benthamiana* plants, the virus-inducible gene *NbKPILP* was discovered. The current work describes the role of the stress-activated cellular factor NtKPILP, a putative homolog of NbKPILP, sharing 95% identity [27,28,29]. Here, we revealed the features and functions of *N. tabacum KPILP*.

Based on the primary structure, NtKPILP belongs to the family of Kunitz peptidase inhibitors. However, the multiple alignment of NtKPILP with KPILPs from other *Nicotiana* species demonstrated that despite the presence of main features characteristic of KPIs, they lack amino Lys or Arg residues in the active center (Figure 1) that are necessary for enzymatic activity [36]. In addition, previously it has been experimentally shown that NbKPILP does not have protease-inhibiting activity [27], so it can be assumed that NtKPILP does not have such activity either, although there is no direct experimental evidence to support this.

*NtKPILP* expression analysis showed the lowest level of expression both in young and mature leaves, while high levels of *NtKPILP* mRNA accumulation were revealed in flowers and roots (Figure 2). Notably, *NtKPILP* is actively expressed in seeds; thus, it could not be excluded that it serves as a storage protein. Most KPIs are highly accumulated in seeds as well (e.g., [37,38]). However, the biological role of such distribution is still not clear. KPIs are suggested being storage proteins or participating in seed protection against herbivores [36]. On the other hand, *N. tabacum* KPI with confirmed inhibitory activity, NtKTI1, was hardly detectable in seeds [30], but similar to NtKPILP, it is characterized by a high level of mRNA in roots; moreover, it corresponding mRNA accumulation correlates with plant age both for KPILPs and NtKTI1.

KPILP homolog (the identity to NtKPILP is 95%) was identified in another tobacco—*N. glutinosa*—where it was designated as cell death marker 1, NgCDM1. *NgCDM1* was discovered due to the significant accumulation of the corresponding mRNA and encoded protein upon TMV infection, and is associated with areas demonstrating HR, a plant reaction that occurs upon incompatible host–pathogen interactions and leads to restriction of infection spread. In addition, *NgCDM1* was shown to be activated in areas of necrotization caused by *Pseudomonas syringae*, indicating that its expression is likely associated with pathogen-induced cell death [31]. However, the role of NgCDM1 during viral or bacterial infection development was not studied, and there is no data on the expression of genes associated with antiviral defense.

In the current study, we have chosen a pathosystem representing an example of compatible interactions: *N. tabacum* L. (cv. Petit Havana), which does not induce HR upon TMV infection, in contrast to *N. glutinosa*, and the infection spreads systemically throughout the plant. We showed that *NtKPILP*, similar to *NbKPILP* and *NgCDM1*, is a TMV-induced gene. It could be suggested that regardless of the type of interactions—incompatible or compatible—KPILPs perform the same functions: modulate the expression of genes associated with antiviral defense and callose metabolism, but in the case of incompatible interactions, the effects mediated by KPILPs are overlaid with other defense mechanisms, e.g., PTI, resulting in HR induction [39]. It could be speculated that evolutionary KPILPs originated from “true” KPIs, keeping the structural similarity but losing the ability to perform the main KPI function—inhibition of proteases. Instead, KPILPs gained novel features enabling them to play an important role in various stresses.

Viral infection causes multiple changes in host plant cells, leading to the induction or suppression of numerous genes’ expression. Cellular factors encoded by these genes can have either proviral or antiviral activity, i.e., they could serve as susceptibility factors or be responsible for plant tolerance or resistance. The altered pattern of gene expression in response to the viral infection leads to significant modifications of many intracellular systems. In particular, chloroplasts, which play an important role in plant development, growth, and defense processes [40], are targeted by viral proteins [41,42]. In addition to the photosynthetic function, chloroplasts harbor many metabolic pathways, the products of which determine the physiological status of the cell and are included in the signaling pathways from plastids to the nucleus. Viruses could affect cellular metabolism via interfering with chloroplasts’ functioning, suppressing defense reactions and thereby creating favorable conditions for effective reproduction and systemic spread throughout the plant. Hypothetically, one of the successful strategies of viral infection could be based on the “switching off” photosynthesis-associated nuclear-encoded genes (PhANG) in leaves by activating the expression of some regulatory genes that are “silent” in leaves but active in roots. Such an activation would lead to the suppression of PhANGs involved in antiviral defense and to the interference with immune response triggered by operational retrograde signals from chloroplasts. Since we have shown that *NtKPILP*, similar to *NbKPILP*, is highly expressed in roots (Figure 2), we evaluated *NtKPILP*’s effect on the expression of several PhANGs regulated by CRS: *LHCB21*, *RBCSIA*, and *HEMAI*. Additionally, we analyzed the expression of *RCA* and *AtpC*, the products of which are involved in the defense response against TMV (Figure 7). For this analysis, we used *N. tabacum* plants with *NtKPILP* knockdown, obtained through virus-induced gene silencing, as it was performed earlier [28]. Activated expression of *NtKPILP* in the group of PVX-infected plants led to the suppression of the above-mentioned PhANGs, while upon *NtKPILP* suppression, on the contrary, their expression was increased. Thus, it can be concluded that, similar to NbKPILP, NtKPILP is involved in the regulation of genes responsible for the transmission of retrograde signals from chloroplasts.

Another NtKPILP function is associated with the regulation of intercellular transport via a callose-dependent mechanism: *NtKPILP* knockdown leads to a decrease in the efficiency of intercellular transport, which correlates with the increased PD callose level. Thus, we concluded that NtKPILP is a positive regulator of intercellular transport. It is possible that the presence of a signal peptide, characteristic of KPILPs from different species (NgCDM1, NbKPILP) [27,31], suggests that NtKPILP undergoes a traditional pathway of intracellular secretion and is possibly localized in the cell wall and/or in the plasmodesmata region. Since the localization of NtKPILP in the cell has not been studied in detail yet, the mechanism underlying its ability to affect PD callose level remains to be elucidated. It could be suggested that NtKPILP regulates the activity of the enzymes participating in the callose metabolism in the cell wall or during the intracellular transport. In addition, NtKPILP could affect PD permeability indirectly, modulating CRS transmission or regulating the expression of genes associated with PD control, e.g., β-1,3-glucanase. Moreover, the reverse correlation between the level of *BG* mRNA and PD callose depositions was revealed upon *NtKPILP* knockdown. Changes in the pattern of retrograde signaling and PD-regulating gene expression could lead to suppression of antiviral defense responses, degradation of callose, and activation of intercellular transport.

## 4. Materials and Methods

### 4.1. Plant Growth Conditions

*Nicotiana tabacum* (cv. Petit Havana) plants were grown in the soil in a controlled environment chamber under a 16 h/8 h day/night cycle.

### 4.2. Agroinfiltration

*Agrobacterium tumefaciens* strain GV3101 was transformed with individual binary vectors and grown at 28 °C in LB medium supplemented with 50 mg/L rifampicin, 25 mg/L gentamycin, and 50 mg/L kanamycin. Bacterial overnight culture was diluted with buffer containing 10 mM MES (pH 5.5) and 10 mM MgSO_4_ and adjusted to a final OD_600_ of 0.01 (agrobacteria containing pPVX or pPVX(frKPILP) plasmid) and OD_600_ of 0.005 for agrobacterium carrying 35S-2×GFP plasmid. Agroinfiltration was performed on fully expanded *N. tabacum* leaves attached to the intact plant. A bacterial suspension was infiltrated into the leaf tissue using a 2mL syringe. After that, the plants were incubated in greenhouse conditions.

### 4.3. Plant Inoculation for Systemic Infection

*N. tabacum* plants were inoculated with pPVX or pPVX(frKPILP) by agroinfiltration of the lower leaves, and in 10–14 days, the systemic PVX infection was detected in the upper leaves. To induce TMV systemic infection, lower leaves of *N. tabacum* plants were inoculated with 300 µg/mL suspension of virus particles in the presence of celite using a brush.

### 4.4. GFP Imaging and Quantification

GFP-containing cell clusters were visualized using an AxioVert 200M fluorescent microscope (Carl Zeiss, Jena, Germany) equipped with an AxioCam MRc digital camera. The excitation and detection wavelengths for GFP were 487 nm and 525 nm, respectively. The lower epidermal cells were analyzed 48 h after agroinfiltration with 35S-2×GFP. Not less than 200 cell clusters per one infiltration area were analyzed, and not less than three biological repeats per experiment. Three experiments were performed.

### 4.5. Callose Staining and Quantification

To visualize PD-located callose, the *N. tabacum* leaves were infiltrated with aniline blue solution (0.1% aniline blue (Sigma Aldrich, Burlington, VT, USA) in 0.01 M K_3_PO_4_ at pH 12). Then, the leaves were incubated in the dark at room temperature for 15 min before imaging using a Nikon C2 laser scanning confocal microscope (Nikon, Tokyo, Japan). The excitation and emission wavelengths for aniline-blue-stained callose were 403 nm and 447 nm, respectively. Four biological repeats were performed for each group; no less than eight areas from each leaf were analyzed, resulting in at least 1000 fluorescent dots in total quantified. Quantification of callose fluorescence intensity was performed as described by Zavaliev and Epel [33] and using the ImageJ software, version 1.47v [43].

### 4.6. NtKPILP Identification and Verification

To amplify the coding region of the *KPILP* from *N. tabacum*, the following primers were selected based on the sequence retrieved from the SolGenomics database (https://solgenomics.net/organism/Nicotiana_tabacum/genome, accessed 15 July 2022): “NtKPILP_fwd” ATGAAGATCATATCAAGGATTTTATTG and “NtKPILP_rev” TTAAGCCTTTTTGAACACAATC. The coding region of NtKPILP was amplified on the cDNA template, which was obtained using an oligo(dT) primer and total RNA isolated from *N. tabacum* leaves. To identify the NtKPILP sequence, the PCR product was cloned into a pKanT vector (Evrogen, Moscow, Russia), and several clones were subjected to Sanger sequencing. The sequence was a complete match to the unannotated sequence from *N. tabacum*. It was designated as NtKPILP and deposited in GeneBank, accession number PQ664906.

### 4.7. Quantitative Real-Time PCR (qRT-PCR) Analysis of Transcript Concentrations

Total RNA was extracted from plant tissues using the ExtractRNA reagent (Evrogen, Moscow, Russia) according to the manufacturer’s instructions. For first-strand cDNA synthesis, random hexamers and oligo(dT) primer were added to 2 µg of total RNA, and reverse transcription was performed using Magnus reverse transcriptase (Evrogen, Moscow, Russia) according to the manufacturer’s protocol. Quantitative real-time PCR was carried out using the iCycler iQ real-time PCR detection system (Bio-Rad, Hercules, CA, USA). Reference gene was detected using the primers to the protein phosphatase 2A gene (PP2A). The target genes were detected using sequence-specific primers and Eva Green master mix (Syntol, Moscow, Russia) according to the manufacturer’s instructions. Primers used for qRT-PCR are listed in Table S1. Each sample was run three times, and a non-template control was added to each run. A minimum of five biological replicates was performed. The results of qRT-PCR were evaluated using the Pfaffl algorithm [44].

### 4.8. Statistical Analysis

The data was analyzed either with a paired two-tailed Student’s *t*-test or with one-way ANOVA, as indicated in figure captions. The significance of the difference between groups was assessed using Tukey honestly significant difference (HSD) test at *p* < 0.05 level or Student’s *t*-test. In all histograms, the y-axis error bars represent the standard error of the mean values.

## Data Availability

The original contributions presented in the study are included in the article/Supplementary Materials.

## Supplementary Materials

The following supporting information can be downloaded at: https://www.mdpi.com/article/10.3390/plants14192955/s1, Figure S1: Representative photographs of different parts of *N. tabacum*; Figure S2: Relative amount of CRS and antiviral defense marker genes mRNA in leaves of plants incubated in darkness; Figure S3: PVX-based vector for *NtKPILP* downregulation; Figure S4: Relative levels of *PVX* RNA in the pPVX- or pPVX(frKPILP)-infected plants; Figure S5: Fluorescent images of aniline blue stained callose in leaf epidermal cells; Table S1: Oligonucleotides used for qRT-PCR.
