# Supplementary material for: Nicotiana tabacum Kunitz Peptidase Inhibitor-like Protein Regulates Intercellular Transport
Source: Plants (Basel). 2025 Sep 23;14(19):2955. doi: 10.3390/plants14192955 (PMC12526089; doi:10.3390/plants14192955)
Supplement: Supplementary file 1 [file plants-14-02955-s001.zip › plants-3829182-supplementary.pdf]

Supplementary material

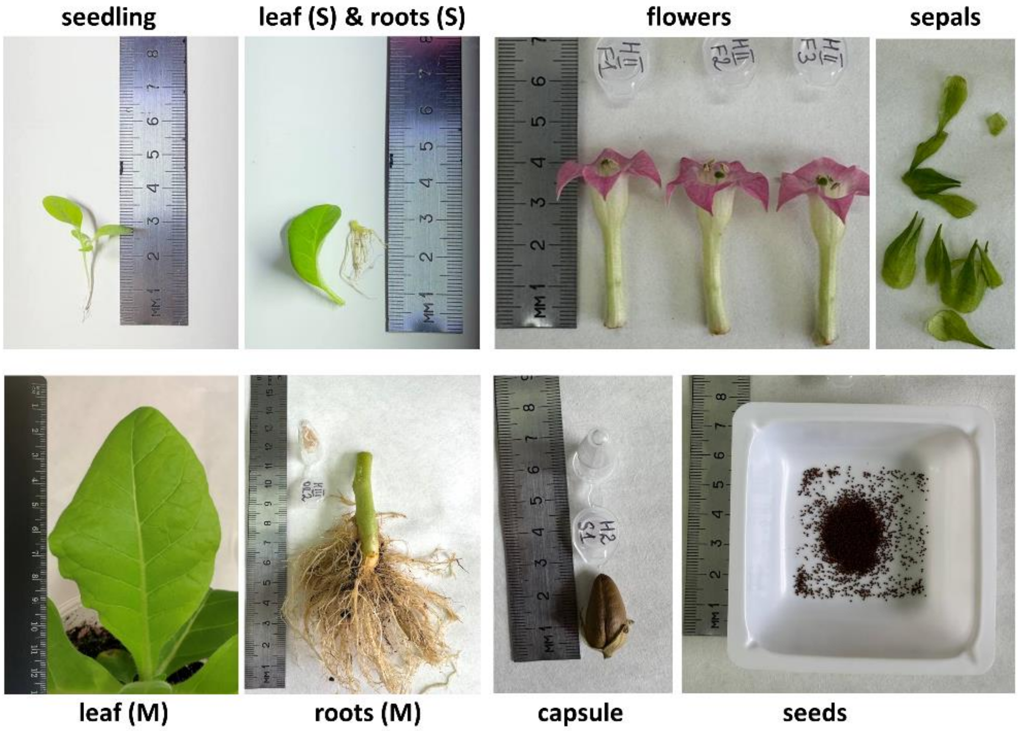

Figure S1. Representative photographs of different parts of *N. tabacum* (cv. Petit Havana) plants taken for the *NtKPILP* mRNA level assessment. Leaves and roots were harvested from 5-6-week-old plants (M, mature plants) or seedlings (S).

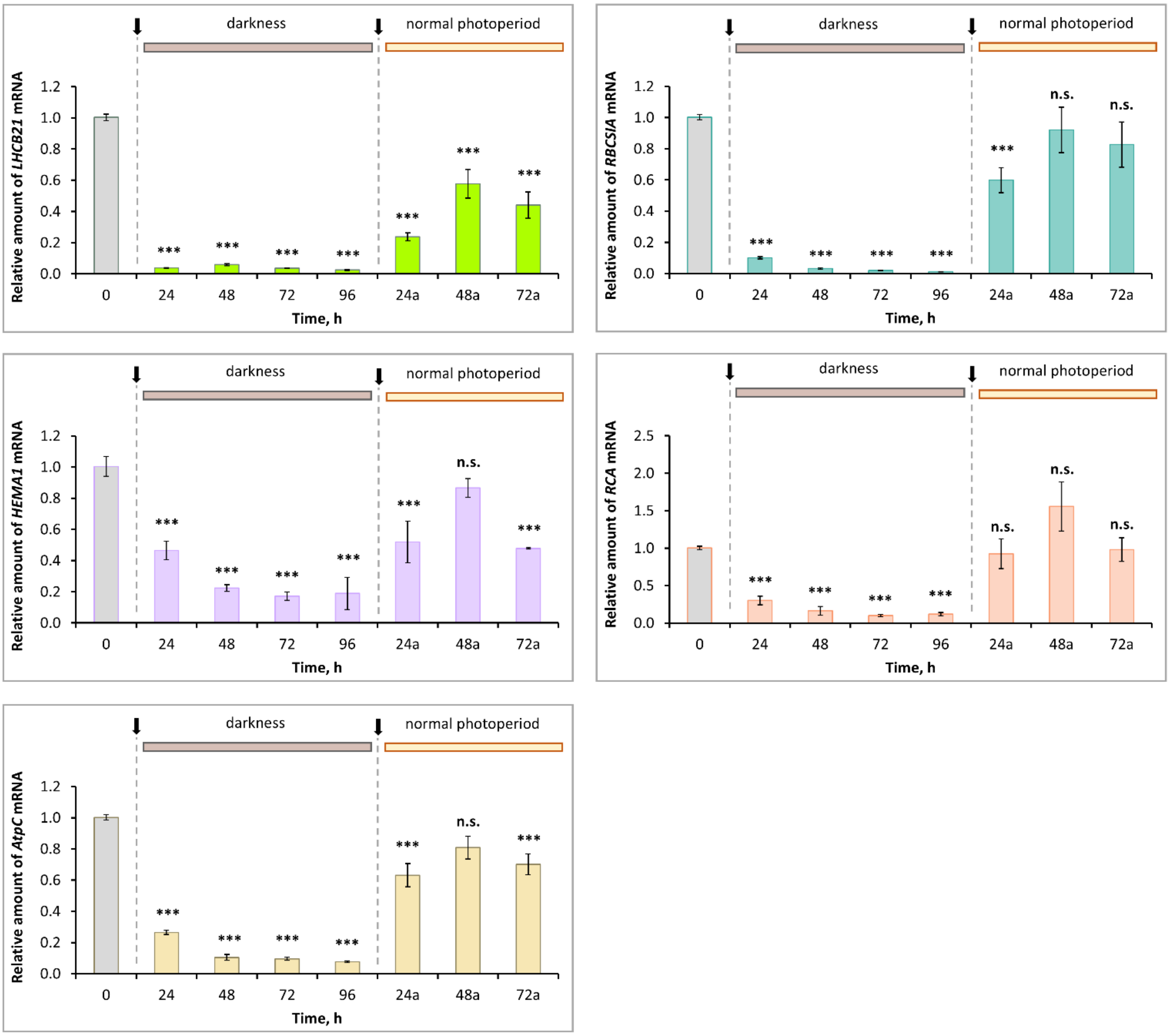

Figure S2. Relative amount of mRNA for CRS and antiviral defense marker genes in leaves of plants incubated in darkness or returned to normal light/dark photoperiod (see Fig. 3A) as determined by qRT-PCR. Index “a” designates the timepoints after plants were returned to the normal photoperiod conditions. Mean values ± SE are presented. The level of mRNA accumulation for each gene at “0” time point was taken as 1. Three independent experiments with three biological repeats each were performed. Paired two-tailed Student’s t-test was applied to assess statistical significance of difference between the samples harvested at “0” time point and other time points, *: $p < 0.05$, ***: $p < 0.001$, n.s., not significant.

```
NbKPILPvigs  1    TTCAAGTGCAGGGCTAGTAATTGACGAAAATGAAGACATAAATATAAAATTTGCAGCACC  60
                  |||||||||||| |||||||||||||||||||||||||||||||||||||||||||||||
NtKPILP      261  TTCAAGTGCAGGACTAGTAATTGACGAAAATGAAGACATAAATATAAAATTTGCAGCACC  320

NbKPILPvigs  61   AAGGTACGTGTCGATTTGCAATAAATCCACTGTTTGGAAAATTGAAGATGGGTTTGTGAG  120
                  |||||||||||| ||||||||||||||||||||||||||||||   || |||||||| |
NtKPILP      321  AAGGTACGTGTCAATTTGCAATAAATCCACTGTTTGGAAAATT---GACGGGTTTGTCAC  377

NbKPILPvigs  121  CACTGGCGGAATTAAGGGTGGGTCAGAAAATGGGACGGCCACAAGTTTGTTTACGATTCA  180
                  |||||||||||||||||||||| | |||||||| ||||||||||||||||||||||||||
NtKPILP      378  CACTGGCGGAATTAAGGGTGGGACTGAAAATGGTACGGCCACAAGTTTGTTTACGATTCA  437

NbKPILPvigs  181  GAAATATGAAGATGTCTATGCGTTACAGTATTGTCCAAGAGCTACAGGGTGTTCTTTTAT  240
                  ||| |||||||||| ||||||||||||||||||||||||||||||||||||||| |||||
NtKPILP      438  GAAGTATGAAGATGCCTATGCGTTACAGTATTGTCCAAGAGCTACAGGGTGTTCCTTTAT  497

NbKPILPvigs  241  TTGCCCCAGATTGTTGTGTGGGTATATTGGTATTTCACCTGCAGCTAATGGATCGAGGCG  300
                  ||| || ||||||||||||||||||||||||||| |||||||||||||||||||||||||
NtKPILP      498  TTGTCCAAGATTGTTGTGTGGGTATATTGGTATTGCACCTGCAGCTAATGGATCGAGGCG  557

NbKPILPvigs  301  TTTGGCTGTGAATCGTCCAGTTTTCAAGATTGTGTTCAAGAAGG  344
                  ||||||||||||||||||||||||||||||||||||||| ||||
NtKPILP      558  TTTGGCTGTGAATCGTCCAGTTTTCAAGATTGTGTTCAAAAAGG  601
```

Figure S3. PVX-based vector for *NtKPILP* downregulation. Nucleotide sequence alignment of *NbKPILP* fragment from pPVX(frKPILP) vector [28] (designated NbKPILPvigs) with *NtKPILP* coding region. The alignment was performed with BLASTn tool.

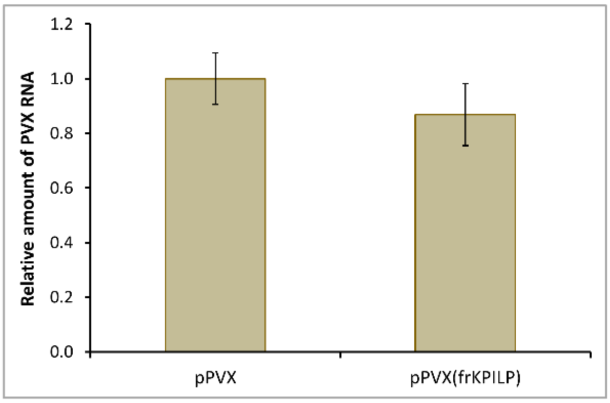

Figure S4. Relative levels of PVX genomic RNA in the pPVX- or pPVX(frKPILP)-infected plants as determined by qRT-PCR. Mean values and SE are obtained from four independent experiments with five biological replicates in each. The level of mRNA accumulation for pPVX-infected plants was taken as 1. Difference between samples from PVX- or pPVX(frKPILP)-infected plants is not significant according paired two-tailed Student`s t-test.

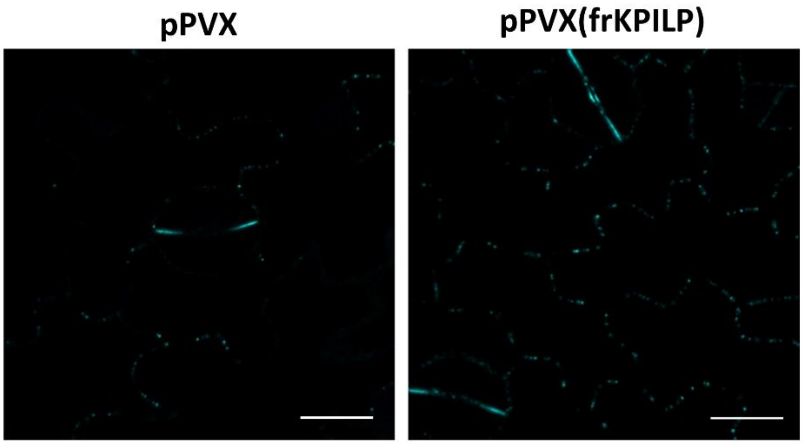

Figure S5. Fluorescent images of aniline blue stained callose in leaf epidermal cells. Leaves of plants with systemic pPVX- or pPVX(frKPILP) infection were infiltrated with aniline blue solution and analyzed with confocal laser scanning microscopy. Bar=20µm.

Table S1. **Oligonucleotides used for qRT-PCR**

Gene	Forward primer	Reverse primer	Accession number
PP2A	ATTGCTGCCTGTGGTTATTAC	ATAGACTGAAGTGCTTGATTGG	MF996339.1*
NtKPILP	CACCACTGGCGGAATTAAGG	GCAATACCAATATACCCACACAAC	PQ664906*
Pol PVX	CTAGGTCTACCCAAGATTA	GTGGTCTCACAGTTTATG	MF405302.1*
LHCB21	AGGACCCAGAGGCATTTG	TAGGACCAGGCGTTGTTG	XM_016635318.2*
RBCS1A	GCTGCCTCATTCCCTGTTTC	CCTGCATGCATTGCACTCTT	Nitab4.5_0009855g0010.1#
HEMA1	ATGTGGGTGCTTGTGTGAAC	AGGCGGTCCTCCTTATTAGC	Nitab4.5_0009708g0010.1#
BG	GATTGTTGTGTCCGAGAGTG	CCAGTTCAGGGTTCTTGTT	EU867448.1*

*GeneBank database https://www.ncbi.nlm.nih.gov/genbank

#SolGenomics database https://solgenomics.net
